# Effects of Hierarchical Unit Culture and Power Distance Orientation on Nurses' Silence Behavior: The Roles of Perceived Futility and Hospital Management Support for Patient Safety

**DOI:** 10.1155/jonm/6564570

**Published:** 2024-11-19

**Authors:** Seung Eun Lee, Jeong Won Lee

**Affiliations:** ^1^Mo-Im KIM Nursing Research Institute, College of Nursing, Yonsei University, Seoul 03722, Republic of Korea; ^2^Department of Business Administration, College of Software and Management, Kyonggi University, Suwon 16227, Republic of Korea

**Keywords:** nurses, organizational culture, patient safety, silence, voice

## Abstract

**Background:** Patient safety is paramount in healthcare, and effective communication is a cornerstone of preventing adverse patient events. Despite nurses' crucial role in improving patient safety, they often keep silent about their concerns. This study investigated links among hierarchical unit culture, nurses' power distance orientation, perception of futility, and silence behavior in healthcare environments. Moreover, we aimed to determine whether nurse-perceived hospital management support for patient safety moderated the association between nurses' perceived futility and silence behavior.

**Methods:** This cross-sectional, correlational study utilized survey data from 730 direct-care nurses working in 88 medical, surgical, or medical–surgical units across 34 hospitals in South Korea. Validated psychometric scales measured the study variables, and data were analyzed using a 2-1-1 type of multilevel structural equation model.

**Results:** Hierarchical unit culture and power distance orientation showed significant positive associations with nurses' perception of futility (*b* = 0.62, *p* < 0.001, and *b* = 0.37, *p* < 0.001, respectively) and subsequently with their silence behavior (*b* = 0.22, *p* < 0.01, and *b* = 0.31, *p* < 0.001, respectively). Futility was found to mediate the relationship of both hierarchical unit culture (indirect effect = 0.222, 95% confidence interval (CI) [0.006, 0.438]) and power distance orientation (indirect effect = 0.132, 95% CI [0.003, 0.261]) to silence behavior. Hospital management support for patient safety significantly moderated the relationship between futility and silence behavior (*b* = 0.04, *p* < 0.05); nurses were less likely to remain silent when they perceived high management support as opposed to low support, regardless of their futility level.

**Conclusion:** Our findings highlight the crucial influence of organizational culture on nurses' silence behavior. The findings also underscore the importance of hospital management support with respect to patient safety. Management support may be necessary to combat nurses' perceived futility and to promote open communication.

## 1. Introduction

Patient safety is fundamental to providing high-quality health services [1]; thus, it should be the principal focus of healthcare professionals [2]. To ensure patient safety, healthcare personnel in potentially unsafe situations should voice their concerns and opinions [3]. For example, when patient safety guidelines are not followed and mistakes or omissions occur, speaking up can prevent adverse patient events [4]. Nurses make up the largest proportion of the healthcare workforce, have frequent close contact with patients, and are usually the first to witness deteriorating conditions in patients [4–6]. When nurses remain silent about safety-related concerns or issues, patient safety is jeopardized [4].

Despite the critical role of nurses in ensuring patient safety, their silence has not been adequately addressed in the literature [4]. Understanding the factors that lead to nurses' silence can help develop strategies to encourage open communication, which is essential for patient safety [7]. Within an organization, silence refers to deliberately withholding information, suggestions, ideas, questions, or concerns regarding issues that could be crucial for the work or the organization [8]. In the context of patient safety, silence refers to the internal behavior of not expressing ideas, information, or opinions that are necessary for keeping patients safe [9]. Employees' silence behaviors are affected by organizational factors, such as the organizational culture, and individual-level psychological factors, such as personal perceptions [10].

Organizational culture is a multifaceted construct encompassing the collective beliefs, values, and norms shared among employees, which significantly shape and influence their actions and behaviors [11]. Employees develop perceptions of the culture around them, which can influence their behavior [12]. A hierarchical culture, characterized by a clear chain of command, respect for authority, and emphasis on maintaining stability and control [13], can affect employee silence [14]. In such cultures, employees may feel constrained or hesitant to voice their opinions, ideas, or concerns because they fear negative consequences for speaking up [6, 15]. In the healthcare context, when personnel fear speaking up, open communication among healthcare team members is hampered [15], negatively affecting patient safety. A recent qualitative study of nurses' speaking-up behaviors [6] reported that a hierarchical culture, described as “inimical to safety,” [16] was one inhibitor of nurses' speaking up for patient safety.

Social exchange theory, which is widely used in the social sciences to explain workplace behavior, relationships, and organizational dynamics [17], posits that social behavior results from an exchange process in which individuals weigh the potential benefits and risks of social relationships [18]. This theory is particularly relevant for understanding why nurses may remain silent in hierarchical cultures. In such environments, nurses may perceive that the risks of speaking up (e.g., potential reprimands or damage to professional relationships) outweigh the benefits, leading to a perception of futility. Futility refers to the belief that voicing concerns or suggestions does not lead to meaningful changes or improvements [19]. In hierarchical cultures, nurses may perceive their input as futile or ineffective because higher-level individuals hold the ultimate authority over decision-making processes [14]. This perception of futility can discourage nurses from engaging in proactive communication, such as speaking up, and inhibit their willingness to contribute ideas, suggestions, or concerns [20]. Thus, we proposed the following hypotheses.

**Hypothesis 1 .**

A hierarchical unit culture is associated with nurses' silence behavior.

**Hypothesis 2 .**

A hierarchical unit culture is associated with nurses' perceived futility.

**Hypothesis 3 .**

Perceived futility mediates the relationship between a hierarchical unit culture and nurses' silence behavior.

Power distance refers to the degree to which individuals accept unequal distributions of status and power within a particular organizational context. Power distance was originally conceptualized as a cultural value [21]. However, as individuals within a society may vary in the extent to which they embrace this value, recent literature has conceptualized power distance as an individual construct rather than a cultural value in an organizational setting. As such, power distance orientation [10, 22, 23] reflects how individuals interpret and accept the distribution of power in an organization [24]. Those with a high-power distance orientation tend to acknowledge and respect hierarchical status differences within their organization. Consequently, they often maintain a distance from organizational superiors, they readily accept decisions from those in higher positions, and they refrain from suggesting ideas or voicing concerns [25]. Such individuals are also more likely to strictly adhere to their defined roles and refrain from undertaking tasks or initiatives that might be perceived as overstepping organizational boundaries. In addition, they may suppress urges to voice disagreement because of fear of repercussions or the belief that disagreeing would be futile [25], which further contributes to silence behavior. In contrast, employees with a low-power distance orientation often downplay the significance of hierarchical distinctions. They tend to believe that their contributions are essential to the organization; consequently, they are more likely to express their concerns, ideas, and suggestions [26]. Employees with a low-power distance orientation may also perceive less futility in speaking up because they believe that their input is valued and can influence organizational outcomes. Given these observations, we proposed the following hypotheses.

**Hypothesis 4 .**

Power distance orientation is related to nurses' silence behavior.

**Hypothesis 5 .**

Power distance orientation is related to nurses' perceived futility.

**Hypothesis 6 .**

Perceived futility mediates the relationship between power distance orientation and nurses' silence behavior.

Management support plays a crucial role in shaping nurses' behavior. For example, one study found that hospital management support for patient safety had a significant impact on nurses' speaking-up behavior [27]. Such support can take the form of proactive management efforts, such as allocating adequate patient care resources and implementing policies that prioritize patient safety [27]. Active implementation of safety-related resources and policies demonstrates that patient safety holds a prominent position among the organization's priorities [28]. According to social exchange theory [17], high levels of management support may enhance the perceived benefits and reduce the perceived risks of speaking up. In other words, when nurses perceive high levels of management support for patient safety in the form of resources and policies, these perceptions may mitigate the impact of perceived futility on their tendency to remain silent [27]. Therefore, we proposed the following hypothesis:

**Hypothesis 7 .**

Hospital management support for patient safety moderates the relationship between nurses' perceptions of futility and their silence behavior.

**Figure 1:**
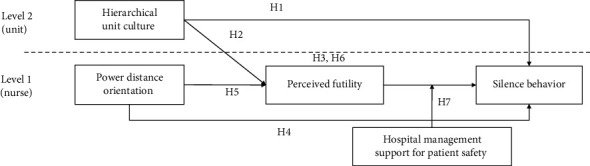
Hypothesized model.

There is limited literature on the complex relationships among a hierarchical unit culture, a power distance orientation, and the perception of futility that contribute to nurses' reluctance to speak up about patient safety. Although some studies [29–32] linked these variables, the results did not fully explore their dynamics within healthcare environments. Furthermore, previous studies failed to adopt a multilevel perspective that considers the characteristics of employees within organizations, particularly in the healthcare context. Moreover, there is limited understanding of the mechanisms and circumstances under which these factors affect nurses' silence behavior. Our study aimed to address these gaps in the literature by adopting a multilevel perspective that considers both organizational and individual characteristics. We investigated the connections between hierarchical unit culture, nurses' power distance orientation, perception of futility, and silence behavior in hospital settings. We aimed to determine whether nurse-perceived hospital management support for patient safety moderates the association between perceived futility and silence behavior, thus providing a comprehensive understanding of these dynamics.  Figure 1 illustrates the hypothesized model.

## 2. Materials and Methods

### 2.1. Study Design, Sample, and Setting

This study was a cross-sectional, correlational study forming part of a larger research project aimed at investigating factors related to patient and workplace safety in healthcare organizations. The original study included 34 out of 149 general hospitals with more than 300 beds located across seven metropolitan areas and nine provinces in South Korea. While random sampling was not feasible, we aimed to achieve a representative sample of nurses by selecting hospitals based on the information available from the Health Insurance Review of Korea website [33]. In the original study, the inclusion criteria for participants were staff nurses with at least 6 months of clinical experience working in general and specialty units within the selected hospitals. The exclusion criteria were nurses in managerial positions or those working only temporarily in the hospitals. Data were collected via an online survey distributed to eligible nurses from February to June 2022. The original study included 1255 participants with a response rate of 72.8%. The detailed sampling methods are in published literature [34].

For this study, we used data from 730 registered nurses who provided direct care in general units. Traditional conventions for determining a multilevel sample size suggest having 30 to 50 groups, each with 30 individuals (the 30-30 rule) [35]; however, studies have often included as few as 1–3 participants per group [36]. Additionally, recent literature suggests that multilevel models can be effectively estimated with smaller group sizes, provided that there is a sufficient number of groups [37]. Our final sample consisted of 730 nurses across 88 units, with an average of 8.3 nurses per unit. Therefore, the sample size met the criteria for conducting a multilevel analysis.

### 2.2. Ethics Statement

This study was approved by the Yonsei University Health System Ethical Review Board (#4-2023-0048). On behalf of the research team, the hospitals sent invitation e-mails to nurses that were eligible to participate in the study. A survey link was sent electronically. Although written consent was not obtained from the participants because of the nature of the online survey, ethical guidelines were strictly followed. Before participating, all respondents were required to acknowledge and agree to their participation by clicking on the checkboxes on the survey's landing page. These checkboxes served as an electronic consent form that provided information on the study's objectives and methods, possible risks and benefits of participating in the study, the voluntary aspect of participation, confidentiality of responses, and secure handling of the data collected. The participants were informed of their right to withdraw from the study at any time without consequence. A gift card worth approximately 15 USD was provided as compensation for participating in the study.

### 2.3. Measures

The study variables included unit-level (hierarchical culture) and individual-level (power distance orientation, perceived futility, silence behavior, perceived hospital management support for patient safety, and demographic information) variables. The scales used to measure these variables were developed and validated in previous studies. All item responses used a 5-point Likert scale ranging from 1 (*strongly disagree*) to 5 (*strongly agree*).

*Hierarchical unit culture* was measured using a five-item scale developed by Han [38] that demonstrated good psychometric properties with Korean nurses [38]. To obtain scores for hierarchical culture at the unit level, an average score was computed for each nurse and then aggregated to produce a unit-level mean score for each unit. We used the intraclass correlation coefficient (ICC) (1), ICC (2), and interrater agreement (Rwg) to confirm the suitability of aggregating individual-level data to the unit level. The ICC (1) for hierarchical culture was 0.15, and the ICC (2), which refers to the reliability of the aggregated data at the unit level, was 0.70, indicating the consistency of responses within each unit. In this study, the ICC values indicated that the measures of hierarchical culture reflected a shared perception among nurses within the same unit. The mean Rwg was 0.83, reflecting the level of interrater agreement in hierarchical culture ratings among nurses in the same unit. Thus, the aggregation of data at the unit level was justified [39, 40]. For the study sample, Cronbach's alpha coefficient for the aggregated scale was 0.72, and exploratory factor analysis confirmed a one-factor model. Higher scores indicate that a unit has a stronger hierarchical culture.

*Power distance orientation* was measured using a five-item scale developed by Yoo, Donthu, and Lenartowicz [41] to assess perceptions of power distance at the individual level. The measure demonstrated good reliability and construct validity in Yoo, Donthu, and Lenartowicz's [41] study with the Korean population. In the present study, Cronbach's alpha coefficient for the scale was 0.73, and exploratory factor analysis confirmed a one-factor model. Consequently, mean scores were calculated, with higher scores indicating a high-power distance orientation.

*Perceived futility* was measured using a three-item scale developed by Burris, Detert, and Chiaburu [42]. Mean scores were calculated, with higher scores indicating higher levels of perceived futility. Burris, Detert, and Chiaburu [42] reported a Cronbach's alpha coefficient of 0.91 for the scale. Cronbach's alpha was 0.78 in the present study. Exploratory factor analysis confirmed a one-factor model.

*Silence behavior* was assessed using a five-item measure developed by Tangirala and Ramanujam [43] to assess nurses' silence behavior regarding patient safety. The measure demonstrated good psychometric properties in a previous Korean study [4]. Mean scores were calculated, with higher scores indicating more silence behavior. In the current study, Cronbach's alpha coefficient for the scale was 0.82 and exploratory factor analysis confirmed a one-factor model.

*Hospital management support for patient safety* was measured using the Hospital Management Support for Patient Safety subscale of the Korean version of the Hospital Survey on Patient Safety Culture [44]. The Cronbach's alpha coefficient for the scale was 0.72 in the present study, and exploratory factor analysis confirmed a one-factor model. Consequently, mean scores were calculated, with higher scores indicating higher levels of management support for patient safety.

### 2.4. Analytical Approach

Before testing the hypotheses, we conducted preliminary analyses. Descriptive statistics and correlations were calculated for all the study variables, and exploratory factor analysis was performed to confirm the validity of the scales. We then tested our hypotheses by performing a multilevel analysis, because the hypotheses and data had a multilevel (individual- and unit-level) structure. Multilevel analysis is a useful method for analyzing data structured into higher-level groups (hospital units) and lower-level individuals (nurses). This method assumes that the individual independence of a unit is maintained at the unit level, whereas the member within a unit has a unique influence on each unit. Because we aimed to examine the mediating effect of perceived futility, multilevel structural equation modeling (MSEM) was selected as an appropriate method. Unlike traditional multilevel modeling methods, MSEM offers advantages when examining multilevel mediation effects. The components of the indirect effects at both the between-group and within-group levels can be estimated independently, without employing the group-mean-centering technique. Instead, a latent variable approach is used to deliver an accurate estimate of the effects occurring at the between-group level [45]. Thus, the hypotheses were tested using MSEM and the Mplus 8.3 program. A bootstrapping test was performed to assess whether the indirect effects were statistically significant.

To confirm the appropriateness of the multilevel analysis, we estimated the proportion of variance in our Level 1 variables at both the within and between levels of analysis by calculating the ICC values. The ICC values for perceived futility and silence behavior were 0.06 and 0.01, respectively. Studies have suggested that if an ICC value exists, the possibility of a Type I error increases [37, 46]; thus, it is desirable to proceed with a multilevel analysis that considers group effects. As described above, individual hierarchical culture scores were aggregated at the unit level, whereas other variables remained at the individual level. Lastly, in the multilevel analyses, we adjusted for individual characteristics associated with variations in perceived futility and silence behavior, namely, the nurses' unit and hospital tenure in years [47].

## 3. Results

### 3.1. Sample Characteristics

As shown in Table 1, the participants were 730 nurses working in 88 medical, surgical, or combined medical–surgical units in 34 hospitals. The average number of participating nurses per unit was 8.3 and ranged from 5 to 16. The participants had an average age of 30.8 (SD = 6.1) years and most were women (98.2%, *n* = 717). The mean nursing experience was 7.2 (SD = 6.0) years, with an average of 6.4 (SD = 5.8) years working in the hospital and 3.7 (SD = 3.0) years working in the unit. Most participants (91.8%, *n* = 670) had at least a bachelor's degree.

Table 2 provides the descriptive statistics for the study variables and results of the correlation analysis. Hierarchical unit culture was not significantly correlated with power distance orientation. As for the other hypothesized relationships, hierarchical unit culture demonstrated a significant positive correlation with perceived futility (*r* = 0.19, *p* < 0.01) and silence behavior (*r* = 0.09, *p* < 0.05). Power distance orientation also showed a significant positive correlation with perceived futility (*r* = 0.29, *p* < 0.01) and silence behavior (*r* = 0.30, *p* < 0.01). Furthermore, perceived futility demonstrated a significant positive correlation with silence behavior (*r* = 0.42, *p* < 0.01). Lastly, hospital management support for patient safety showed a significant negative correlation with nurses' silence behavior (*r* = −0.21, *p* < 0.01).

### 3.2. Hypothesis Testing

Before testing the hypotheses, we conducted exploratory factor analysis. The results using principal component analysis confirmed the validity of multi-item variables. All items loaded significantly on their respective factors, supporting a five-factor model with factor loadings of 0.52 or higher. Next, we tested the study hypotheses and the results are presented in Table 3. We used MSEM to analyze the data structured into unit-level groups and individual nurses. MSEM also enabled the examination of complex relationships, including mediation effects, at multiple levels. Thus, for hypothesis testing, a 2-1-1 multilevel mediation model was specified and analyzed [45]. The overall model fit indices were found to be suitable for the data as follows: *χ*^2^(4) = 5.55 (*p* > 0.05), comparative fit index = 0.98, Tucker–Lewis index = 0.94, root mean square error of approximation = 0.04, standardized root mean square residual within = 0.01, and standardized root mean square residual between = 0.27.

As shown in Model 1, hierarchical unit culture was positively associated with silence behavior (*b* = 0.22, *p* < 0.01), supporting Hypothesis 1. Also, as shown in Model 4, hierarchical unit culture had a positive relationship with perceived futility (*b* = 0.62, *p* < 0.001), supporting Hypothesis 2. Hypothesis 3 proposed that futility would mediate the relationship between hierarchical unit culture and silence behavior. In Model 2, futility was added to the analysis after hierarchical unit culture and power distance orientation were included. As shown in Model 2, the positive relationship between futility and silence behavior was significant (*b* = 0.27, *p* < 0.001). Next, to assess whether indirect effects were statistically significant, bootstrapping tests were performed. As shown in Table 4, the indirect effect of hierarchical unit culture via futility did not include zero between the upper and lower limits of the 95% confidence interval. Thus, Hypothesis 3 was supported. Additionally, hierarchical unit culture was nonsignificant (*b* = −0.00, *p* > 0.05), indicating that futility fully mediated the effect of hierarchical unit culture on silence behavior.

Hypothesis 4 posited a positive relationship between nurses' power distance orientation and silence behavior. This hypothesis was supported by the findings in Model 1, where power distance orientation had a significant positive effect on silence behavior (*b* = 0.31, *p* < 0.001). Hypothesis 5 posited a positive link between power distance orientation and perceived futility. This hypothesis was supported in Model 4, where a positive association was observed between power distance orientation and futility (*b* = 0.37, *p* < 0.001). Hypothesis 6 proposed that futility would mediate the relationship between power distance orientation and silence behavior. This hypothesis was also supported. The indirect effect of power distance orientation on silence behavior through futility was significant, as the 95% confidence interval did not include zero (Table 4). In Model 3, power distance orientation was significant (*b* = 0.21, *p* < 0.001), indicating futility partially mediated the relationship.

**Figure 2:**
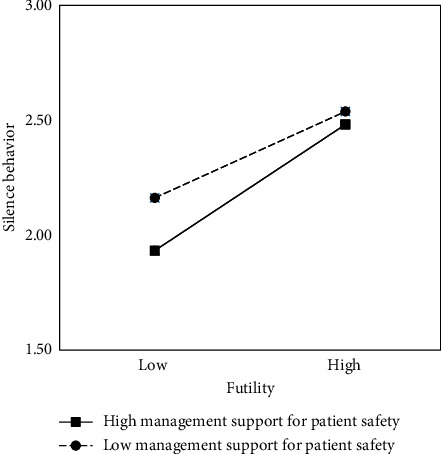
Graph of the interaction effect.

Hypothesis 7 posited that the influence of perceived futility on silence behavior would be moderated by hospital management support for patient safety. This hypothesis was supported in Model 3 of Table 3, where the interaction term (futility × hospital management support for patient safety) was significant (*b* = 0.04, *p* < 0.05). In addition, although not initially hypothesized, hospital management support for patient safety demonstrated a significant negative association with silence behavior (*b* = −0.18, *p* < 0.001). The interaction effect was then plotted as shown in Figure 2, and the graph showed that regardless of the level of perceived futility, nurses were less likely to remain silent when hospital management support for patient safety was high compared to when it was low. This compensatory interaction effect accounts for situations in which a high level of a single factor (i.e., management support or futility) is sufficient to bring about a high or low level of work outcome (i.e., silence behavior). In other words, a high level of hospital management support for patient safety compensates for favorable conditions for silence (i.e., high futility), while a low level of futility compensates for favorable conditions for silence (i.e., low management support). Thus, Hypothesis 7 was supported.

**Figure 3:**
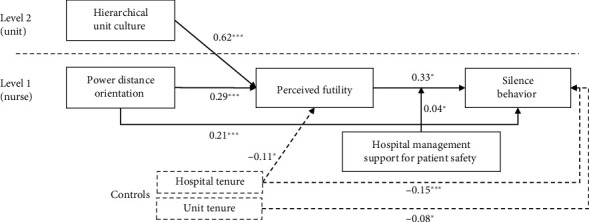
Results of the multilevel structural equation modeling. Only significant paths with standardized coefficients are reported. The dashed lines indicate relationships with the control variables. ⁣^∗^*p* < 0.05, ⁣^∗∗^*p* < 0.01, ⁣^∗∗∗^*p* < 0.001.

Figure 3 illustrates the relationships among the variables using standardized coefficients. Hierarchical unit culture had a positive effect on perceived futility (*β* = 0.62, *p* < 0.001). Power distance orientation also significantly predicted perceived futility (*β* = 0.29, *p* < 0.001). Perceived futility, in turn, positively influenced silence behavior (*β* = 0.33, *p* < 0.05). In general, our findings suggest that a hierarchical unit culture and power distance orientation are critical predictors of nurses' perceived futility, and futility leads to silence behavior. However, robust hospital management support for patient safety can reduce silence behavior, underscoring the importance of supportive organizational practices.

## 4. Discussion

This study provides a multilevel analysis of the interplay between organizational culture, individual perceptions, and nurses' silence behavior. By examining the relationships between hierarchical unit culture, power distance orientation, perceived futility, and silence behavior, our findings revealed that a hierarchical unit culture and power distance orientation were positively associated with the perception of futility among nurses, which, in turn, contributed to their silence behavior. Furthermore, this study offers new insights by demonstrating that hospital management support for patient safety can mitigate the constraints of a hierarchical culture and reduce silence behavior, irrespective of futility levels. These findings echo prior studies highlighting the detrimental effects of hierarchical environments on open communication within both medical [15] and broader organizational settings [48], and they contribute to practical advances in nursing management.

Consistent with results of previous studies in healthcare [49, 50] and nursing [6], our findings indicated that a hierarchical culture often hinders employees from speaking up. Nurses, who are positioned lower in hospital hierarchies [51], may believe that their opinions and concerns will be disregarded [15]. The hierarchical culture inherent in healthcare can create situations in which nurses feel discouraged from speaking up, thereby impeding open communication among different levels of healthcare personnel. In addition to organizational hierarchies, our findings revealed that individuals' orientation toward power distance can affect their silence behavior. Individuals with a high-power distance orientation tend to accept the decision-making authority of their superiors and are less likely to voice their concerns or opinions [10]. Our results suggest that reducing a unit's hierarchical culture and addressing a high-power distance orientation are essential for diminishing nurses' silence behavior.

Silence is particularly problematic among nurses who are usually the first to notice a patient's deterioration or the potential for patient harm [52]. When nurses do not feel empowered to raise concerns, timely interventions can be missed, potentially escalating minor issues into emergencies. Moreover, silence behavior can have a detrimental effect on the morale and mental well-being of the nurses themselves, particularly if they believe that they should have spoken up earlier or intervened in potentially harmful situations [52, 53].

Addressing nurses' silence issues requires management efforts to create an environment where nurses can freely voice their observations of potential risks without fear of repercussions [6, 15]. Nurse managers should recognize the significance of individual nurses' attitudes toward power distance. For nurses inclined toward a high-power distance orientation, nurse managers should carefully consider their leadership behaviors and encourage them to communicate openly [23]. Additionally, as we observed in this study, hospital management support for patient safety plays an essential role in reducing nurses' silence. Proactive steps and demonstrated commitment by management can help create an environment where nurses feel protected and encouraged to raise their concerns. Hospital management support for patient safety includes such actions as developing speaking-up policies [54] and facilitating interdisciplinary team training on nonhierarchical, open communications [51, 55, 56].

Management support implementation presents significant challenges [11] that are often beyond the control of managers, such as resource constraints, varying management priorities, and entrenched hierarchical cultures that hinder the effectiveness of support initiatives [48]. Consequently, a multifaceted approach is necessary that extends beyond a single intervention, such as consistent and visible commitment from top management [57] and ongoing presence and role modeling and mentorship by relational leaders at the unit level [58]. Future research should explore cost-effective strategies to overcome barriers to speaking up for patient safety, particularly the impact of management support on individual, unit, and organization-level outcomes.

This study has several limitations. First, its cross-sectional design precludes the determination of causality because it only captures a single point in time and cannot confirm temporal relationships between the variables. Additionally, the study's reliance on self-reported data may introduce potential response biases such as social desirability and recall bias. Participants, for example, might have overreported or underreported their behaviors or experiences to present themselves in a more favorable light. Additionally, the uniform source of data collection raises concerns regarding common method bias. Future studies could mitigate this bias by incorporating diverse data sources (e.g., peer and manager evaluations) to provide more comprehensive assessments. Furthermore, although we accounted for variables such as nurses' unit and hospital tenure, other confounding factors may have affected the relationships observed. Additionally, the absence of a randomized selection process means that the selected hospitals may not fully represent nurses at all Korean hospitals, thereby affecting the generalizability of our results. Lastly, while the sample size met the criteria for conducting a multilevel analysis, the small sample size in this study may have affected the accuracy of the results.

## 5. Conclusions

The findings substantiate the influence of organizational culture and individual perceptions on nurses' silence behavior. Hierarchical unit culture and power distance orientation emerged as significant predictors of nurses' perception of futility, which, in turn, contributed to their silence behavior. The moderating effect of management support on patient safety is of paramount importance. The results highlighted that even in environments where nurses perceive speaking up as futile, management support can mitigate the risk of nurses being silent. This finding suggests that although the long-term reshaping of organizational culture is vital, immediate benefits can be realized by ensuring that management clearly displays its unwavering commitment to patient safety. Overall, hospital administrators should provide adequate patient care resources to ensure patient safety, and they should foster supportive environments that encourage transparent communication. Such efforts are needed to develop safer, more responsive, and more effective healthcare delivery systems.

## Figures and Tables

**Table 1:** Participants' demographic characteristics (*N* = 730).

Characteristic	*n* (%)	Mean (standard deviation)
Age (years)		30.8 (6.1)
Gender		
Male	13 (1.8)	
Female	717 (98.2)	
Tenure		
Unit (years)		3.7 (3.0)
Hospital (years)		6.4 (5.8)
Employment status		
Permanent	726 (99.5)	
Temporary	4 (0.5)	
Education level		
Diploma	60 (8.2)	
Bachelor's degree or higher	670 (91.8)	
Work unit		
Medical	291 (39.9)	
Surgical	284 (38.9)	
Medical–surgical	155 (21.2)	

**Table 2:** Descriptive statistics and correlational results.

Variable	1	2	3	4	5	6
1. Unit tenure						
2. Hospital tenure	0.38⁣^∗∗^					
3. Hierarchical unit culture	−0.06	−0.09⁣^∗^				
4. Power distance orientation	−0.04	−0.02	0.04			
5. Perceived futility	−0.08⁣^∗^	−0.09⁣^∗^	0.19⁣^∗∗^	0.29⁣^∗∗^		
6. Silence behavior	−0.15⁣^∗∗^	−0.22⁣^∗∗^	0.09⁣^∗^	0.30⁣^∗∗^	0.42⁣^∗∗^	
7. Hospital management support for patient safety	−0.03	0.03	−0.10⁣^∗∗^	−0.07	−0.26⁣^∗∗^	−0.21⁣^∗∗^
Mean	3.31	6.05	3.60	2.18	2.32	2.27
Standard deviation	3.07	5.81	0.22	0.58	0.76	0.61

⁣^∗^*p* < 0.05.

⁣^∗∗^*p* < 0.01.

**Table 3:** Results of the multilevel analysis.

	DV: Silence behavior			DV: Perceived futility
	Model 1	Model 2	Model 3	Model 4
Level 1 variables				
Unit tenure	−0.02⁣^∗∗^ (0.01)	−0.02⁣^∗^ (0.01)	−0.02⁣^∗^ (0.01)	−0.01 (0.01)
Hospital tenure	−0.02⁣^∗∗∗^ (0.00)	−0.02⁣^∗∗∗^ (0.00)	−0.02⁣^∗∗∗^ (0.00)	−0.01⁣^∗^ (0.01)
Power distance orientation	0.31⁣^∗∗∗^ (0.04)	0.21⁣^∗∗∗^ (0.04)	0.21⁣^∗∗∗^ (0.04)	0.37⁣^∗∗∗^ (0.05)
Perceived futility		0.27⁣^∗∗∗^ (0.03)	0.13⁣^∗^ (0.06)	
Hospital management support for patient safety			−0.18⁣^∗∗∗^ (0.05)	
Interaction term			0.04⁣^∗^ (0.02)	
Level 2 variable				
Hierarchical unit culture	0.22⁣^∗∗^ (0.08)	−0.00 (0.13)	−0.04 (0.13)	0.62⁣^∗∗∗^ (0.14)

Within-level residual variance	0.32⁣^∗∗∗^ (0.02)	0.28⁣^∗∗∗^ (0.02)	0.27⁣^∗∗∗^ (0.02)	0.48⁣^∗∗∗^ (0.04)
Between-level residual variance	0.00 (0.01)	0.00 (0.01)	0.00 (0.01)	0.01 (0.01)

*Note:* Level 1 *N* = 730, Level 2 *N* = 88. Unstandardized path coefficients and standard errors (in parentheses) are reported.

Abbreviation: DV = dependent variable.

⁣^∗^*p* < 0.05.

⁣^∗∗^*p* < 0.01.

⁣^∗∗∗^*p* < 0.001.

**Table 4:** Results of significance test of indirect effects.

Route	Boot coefficient	Boot SE	95% confidence interval	
			LLCI	ULCI
Hierarchical unit culture ⟶ perceived futility ⟶ silence behavior	0.222	0.110	0.006	0.438
Power distance orientation ⟶ perceived futility ⟶ silence behavior	0.132	0.066	0.003	0.261

Abbreviations: LLCI = lower level of the confidence interval, SE = standard error, ULCI = upper level of the confidence interval.

## Data Availability

The data that support the findings of this study are available on request from the corresponding author. The data are not publicly available due to privacy or ethical restrictions.
